# Indolent Infection After Lumbar Interbody Fusion: An Under-recognized Cause of Pseudarthrosis, Which Can Be Successfully Treated With Anterior Revision Fusion

**DOI:** 10.5435/JAAOSGlobal-D-21-00259

**Published:** 2022-03-02

**Authors:** Andrew S Zhang, Ellis M. Berns, Davis A. Hartnett, Eren O. Kuris, Alan H. Daniels

**Affiliations:** From the Department of Orthopaedic Surgery, Warren Alpert Medical School of Brown University, Providence, RI (Dr. Zhang, Dr. Kuris, and Dr. Daniels), and Warren Alpert Medical School of Brown University (Mr. Berns and Mr. Hartnett).

## Abstract

**Introduction:**

Bacterial infection is a common etiology for pseudarthrosis after transforaminal lumbar interbody fusion, although it is often difficult to identify because of a delayed presentation and normal laboratory values. The primary goal of this study was to present a series of cases demonstrating patients with infection-related pseudarthrosis successfully managed with anterior revision.

**Methods:**

We retrospectively reviewed patients presenting to a single academic spine center who were found to have evidence of *Cutibacterium acnes* or coagulase-negative *Staphylococcus* infection on routine culturing of lumbar interbody fusion revisions from July 2019 to January 2021. All patients underwent salvage of a transforaminal lumbar interbody fusion pseudarthrosis through an anterior lumbar approach.

**Results:**

A total of six patients managed for pseudarthrosis secondary to suspected infection were eligible for this study (mean age 64.8 years, range 54-70 years; mean body mass index, range 24.5-39.1). Persistent radiculopathy was the primary presenting symptom in all patients with a mean time to revision of 17 months. Coagulase-negative *Staphylococcus* was the primary pathogen, identified from intraoperative samples in 50% of the cases. All patients demonstrated a resolution of symptoms after placement of an anterior lumbar interbody cage, without intraoperative complications, and a subsequent antibiotic regimen.

**Discussion:**

Indolent infection is an under-recognized cause of pseudarthrosis of the lumbar spine. Revision surgery through an anterior lumbar approach, which promotes ease of cage removal and optimized alignment and surface area available for revision fusion, is sufficient to manage pseudarthrosis due to infection.

Transforaminal lumbar interbody fusion (TLIF) and posterior lumbar interbody fusion (PLIF) are common procedures done in the treatment of lumbar degenerative diseases.^1,2^ Pseudarthrosis, a failure of adequate fusion between adjacent vertebral bodies, is a complication seen in 2.6% to 15% of initial posterior fusion procedures, with reoccurrence reported in up to 51% of patients after revision surgery.^3,4^

Patient-related risk factors for pseudarthrosis include diabetes, use of steroids or nonsteroidal anti-inflammatory drugs, and smoking.^5^ However, recent reports have described infection as a common etiology for pseudarthrosis, particularly *Cutibacterium acnes* (C acnes) and coagulase-negative *Staphylococcus* (CoNS).^6,7,8,9^ *C acnes*, formerly known as *Propionibacterium acnes*, is a gram-positive, anaerobic rod bacteria that is native to the hair follicles of the face, neck, and back.^10,11^ Pseudarthrosis secondary to *C acnes* infection is often presumed to be aseptic because of its delayed presentation and often normal infectious laboratory values, including complete blood count, C-reactive protein, and erythrocyte sedimentation rate.^6,7,12,13^ Because of the low virulence and slow growth of *C acnes*, prolonged incubation time, usually 2 weeks at minimum, is required for its identification as the source of fusion failure.^14^

If pseudarthrosis secondary to infected lumbar interbody fusion is suspected, 1 proposed treatment strategy is revision anterior lumbar interbody fusion (ALIF). Advantages of ALIF include avoidance of posterior scar tissue, ease of cage removal, ample exposure for removal of the infected disk material, and full restoration of biomechanical alignment and stability.^15,16^

Multiple recent case series have described successful salvage of TLIF pseudarthrosis with ALIF^17,18^; however, to our knowledge, no patients described with pseudarthrosis secondary to *C acnes* or CoNS infection resolved with ALIF have been reported. At our institution, routine culturing of lumbar interbody fusion revisions led to several cases of indolent infection as a cause of pseudarthrosis. We present a case series describing TLIF pseudarthrosis secondary to *C acnes* and CoNS infection and salvage with anterior revision, débridement, and fusion.

## Methods and Results

This study was a retrospective case series of patients who underwent salvage of a TLIF pseudarthrosis through an anterior lumbar approach, with findings indicating *C acnes* or CoNS infection at a single center between July 2019 and January 2021. A total of six patients managed for pseudarthrosis secondary to suspected infection were eligible for this study; five male and one female patient with a mean age of 64.8 years (range, 54-70) and a mean body mass index of 24.9 (range 24.5-39.1). Of the identified patients, 83% were smokers, 83% had a diagnosis of hypertension, and 50% had a history of abdominal surgery (Table 1). Persistent radiculopathy was the primary presenting symptom in all patients with a mean time to revision of 17 months (range 6-45 months), and all patients underwent placement of a titanium anterior lumbar interbody cage without intraoperative complications (Table 2). CoNS was identified from intraoperative samples in 50% of the cases and *C acnes* in 33%, with one case finding gram-positive cocci, gram-positive rods, and gram-negative rods. All patients were placed on appropriate antibiotic regimens after bacterial identification (Table 3).

**Table 1:** Patient Demographic Data

Patient	Age	Sex	BMI	Smoking (Yes/No)	Diabetes (Yes/No)	HTN (Yes/No)	Prior Abdominal Surgery	Additional Relevant History
1	68	M	28.2	No	No	Yes	None	Remote thyroid cancer
2	69	M	26.1	Yes	No	Yes	None	COPD
3	54	M	39.1	Yes	No	Yes	None	Hemochromatosis and hypothyroidism
4	65	M	29.3	Yes	No	Yes	Sigmoidectomy	
5	63	F	24.5	Yes	No	No	Total abdominal hysterectomy	Chronic bronchitis and hypothyroidism
6	70	M	29.3	Yes	No	Yes	Inguinal hernia	Prostate cancer treated with radiation

BMI = body mass index, COPD = chronic obstructive pulmonary disease, HTN = hypertension

**Table 2:** Patient Surgical Data

Patient	Previous Fusion Levels	Presenting Findings	Second Operation	Time to Revision (mo)	Op Time	Blood Loss (mL)	Cage	Surgical Complications	Radiographic Fusion After ALIF?
1	L2-3; L5-S1	Severe RLE radiculopathy	Revision L5-S1 ALIF and posterior instrumentation	45	4:45	400	Spine art secured lumbar anterior cage	None	Yes
2	L5-S1	Lower back pain and bilateral LE numbness/tingling	L5-S1 ALIF with revision posterior T12-S1	20	4:42	510	Spineart secured lumbar anterior cage	None	TBD
3	L5-S1	Broken posterior rod with back pain and RLE radiculopathy	L5-S1 ALIF	6	4:55	500	Spineart secured lumbar anterior cage	None	TBD
4	L5-S1	Radiographic screw/cage loosening and LLE radiculopathy	L5-S1 ALIF with L2-pelvis revision instrumentation	7	5:42	500	Spineart secured lumbar anterior cage	None	TBD
5	L4-5	Continued lower back pain with bilateral LE numbness	L4-5 and L5-S1 ALIF and L4-pelvis	8	7:38	700	Spineart secured lumbar anterior cage	None	No
6	L3-4, L4-5, L5-1 TLIFs, L2-S1 fusion	Subsidence of L5-S1 and RLE radiculopathy	L5-S1 ALIF and L2-pelvis January 21, 2021	16	8:16	400	Spineart secured lumbar anterior cage	None	TBD

ALIF = anterior lumbar interbody fusion, LE = lower extremity, LLE = left lower extremity, RLE = right lower extremity, TBD = to be determined

**Table 3:** Patient Infection Data

Patient	Identified Bacteria	Length of Stay (d)	Antibiotic Regimen	Treatment Complications
1	Coagulase negative *staphylococcus*	5	8 wk IV vancomycin and oral rifampin and then 6 mo of oral doxycycline and oral rifampin	Oral rifampin discontinued after 4 mo because of nausea
2	Gram-positive cocci, gram-positive rods, and gram-negative rods	7	6 wk IV vancomycin and IV cefepime and then oral minocycline for 6 mo	Cefepime discontinued after 5 wk because of AKI and diffuse rash
3	*Cutibacterium acnes*	3, 2	6 wk IV ceftriaxone and then 5 mo oral doxycycline	*Clostridium difficile* infection
4	*Cutibacterium acnes*	4, 3	6 wk IV ceftriaxone and then 3 mo oral doxycycline	None
5	Coagulase negative *staphylococcus*, *Corynebacterium*	9	1 wk IV vancomycin and then 1 mo oral doxycycline	Doxycycline briefly paused because of diarrhea
6	Coagulase negative *staphylococcus*	6	8 wk IV ceftriaxone and IV vancomycin and oral rifampin and then 6-12 mo oral doxycycline	Oral rifampin discontinued because of a moderate allergic reaction

AKI = acute kidney injury, IV = intravenous

## Case Illustrations

### Case 1

**Figure 1:**
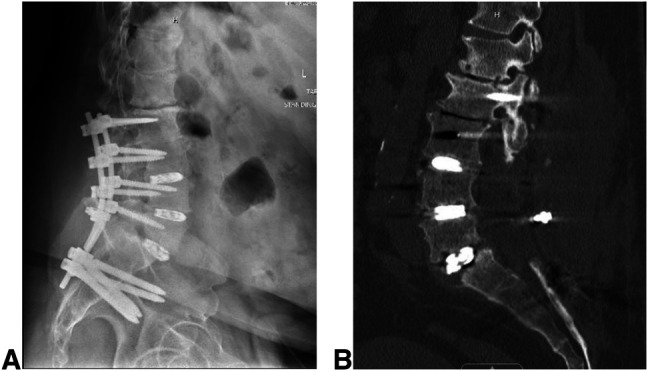
**A**, Lateral radiograph of the lumbar spine demonstrating a broken rod of the previous construct. **B**, Radiograph showing the sagittal cut of a CT scan of the lumbar spine demonstrating pseudarthrosis of L5-S1 with subsidence of a transforaminal lumbar interbody fusion cage.

A 54-year-old man (patient 3) presented with 1 month of persistent and progressive lower back pain and severe right lower extremity radicular symptoms. He previously underwent an L3-L5 decompression and fusion and a L5-S1 TLIF, and most recently underwent a revision L2-pelvis decompression and fusion for nonunion. No cultures were obtained at that time. He was doing well until the sudden onset of sharp back pain while walking, later radiating into his leg. Radiographs and a CT scan of the patient's lumbosacral spine revealed evidence of L5-S1 pseudarthrosis, loss of foraminal height, and a broken rod (Figure 1). The patient had failed conservative management and elected to proceed with revision surgery.

**Figure 2:**
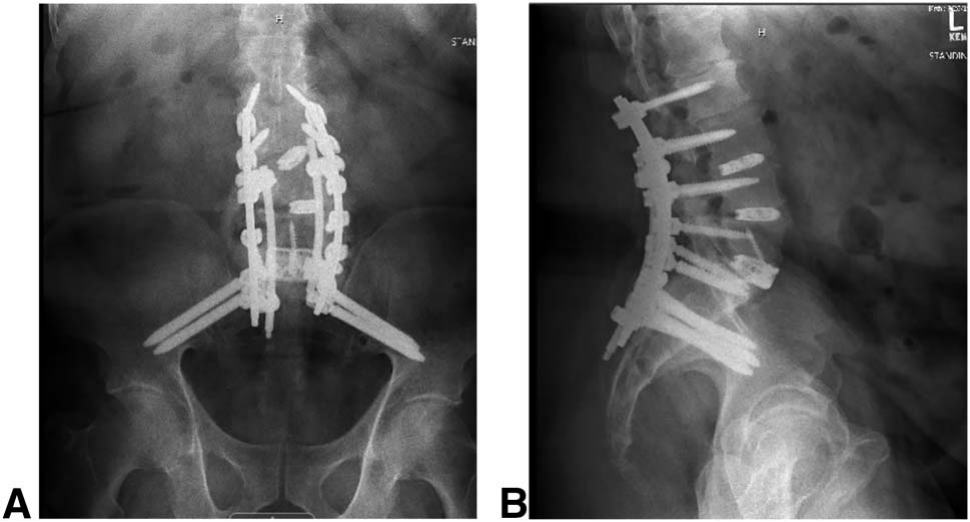
**A**, AP and (**B**) lateral radiographs of the lumbar spine showing replacement of infected L5-S1 transforaminal lumbar interbody fusion with an anterior lumbar interbody fusion cage.

Through a standard anterior approach to the lumbar spine, the L5-S1 disk space was accessed and the previous cage was easily identified, noted to be loose, readily removed, and sent for culture along with specimens of the intervertebral disk. After thorough débridement and end plate preparation, a new titanium ALIF cage packed with highly porous beta-tricalcium phosphate, allograft chips, recombinant human bone morphogenetic protein-2 (rhBMP-2), and vancomycin powder was inserted and secured with screws (Figure 2). Vancomycin powder was placed within the wound and then the incision was closed in multiple layers. The patient was transferred to the postanesthesia care unit before being admitted to the regular floor, where his pain management and mobility progressed appropriately, and he was discharged on oral pain medication on postoperative day 3. Of note, the patient completed 24 hours of cefazolin postoperatively and then transitioned to empiric Augmentin (amoxicillin-clavulanic acid) until cultures resulted. On postoperative day 5, the three intraoperative cultures unanimously grew *C acnes*, and the patient was readmitted for antibiotic therapy and monitoring. Blood cultures were obtained, and the patient was started on intravenous (IV) ceftriaxone. Infectious disease services recommended ceftriaxone for 6 weeks, and he was discharged home again after the placement of a central catheter. The treatment was complicated by a *Clostridium difficile* infection managed with 14 days of oral vancomycin. After ceftriaxone therapy, the patient was transitioned to an oral doxycycline regimen for an additional 3 months, a regimen that was prolonged two additional months because of metatarsal surgery. At his 9-month follow-up, he had completed antibiotic therapy without recurrence of infection and reported markedly improved lower back pain and the absence of radicular symptoms with an increased ability to perform his activities of daily living.

### Case 2

**Figure 3:**
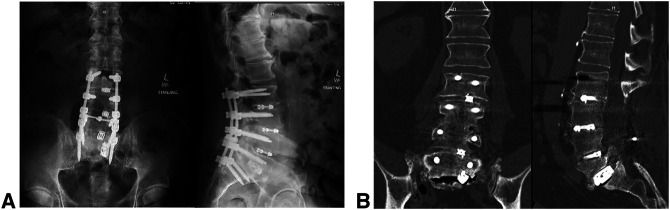
**A**, AP and lateral radiographs and (**B**) coronal and sagittal views of the lumbar spine showing L2-S1 instrumented fusion with L5-S1 pseudarthrosis and a vacuum disk space with cage subsidence.

**Figure 4:**
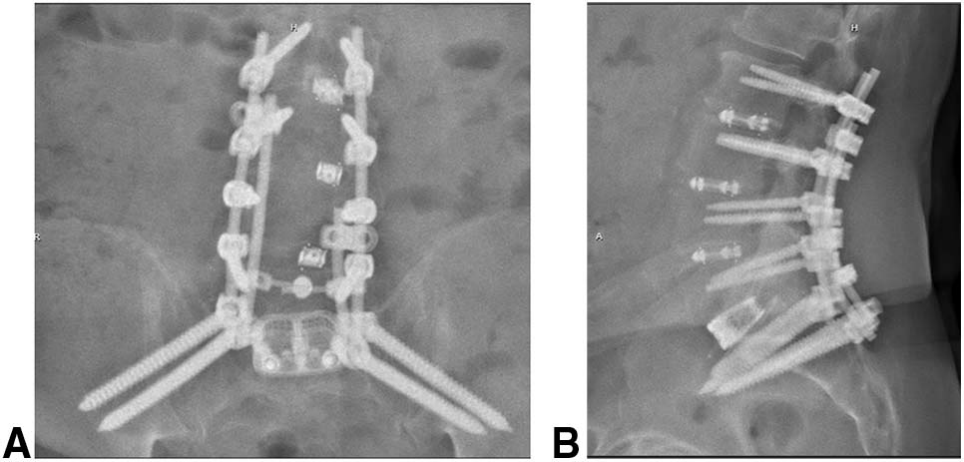
**A**, Postoperative AP and (**B**) lateral radiographs of the lumbar spine showing revision of L5-S1 transforaminal lumbar interbody fusion to anterior lumbar interbody fusion with reduction of spondylolisthesis at that level, and reduction of spondylolisthesis and restoration of foraminal height at L5-S1. There is additional pelvic fixation in the form of bilateral sacral-2-alar-iliac screws.

A 68-year-old man (patient 1) with a history of two lumbar decompression and interbody fusion surgeries continued to experience back pain despite epidural injections. He reported severe disability from persistent lower back pain and right leg radiculopathy. His examination was notable for diminished sensation in the right L5 distribution. Radiographs revealed previous L2-S1 decompression and fusion with interbody support without instrumentation failure or fracture (Figure 3), but CT of the lumbar spine did show L5-S1 pseudarthrosis with interval increased L5-S1 spondylolisthesis and vacuum disk phenomenon and bilaterally loosened S1 pedicle screws. MRI of the lumbar spine also showed a large calcified L5-S1 disk herniation with impingement of the bilateral L5 and S1 nerve roots. Having failed conservative management, the patient underwent a revision L5-S1 ALIF with osteotomy, followed by posterior revision L2-S1 decompression and fusion almost 4 years after the index interbody surgery of L5-S1. Through the anterior approach, the previous L5-S1 TLIF cage was removed and sent for culture. The remainder of the L5-S1 disk space and end plates was prepared, and a titanium ALIF cage packed with highly porous beta-tricalcium phosphate, allograft chips, rhBMP-2, and vancomycin powder was implanted without issue. The patient was then turned prone and underwent revision posterior L2-pelvis instrumented fusion. Screws from L2-L5 were noted to be rigidly implanted in the bone, with a broken screw head at L2, and the decision was made to leave these screws in place. S1 screws were noted to be loose and were replaced, and sacral-2-alar-iliac screws were placed for pelvic fixation (Figure 4). Pseudarthrosis was confirmed at L5-S1, and revision decompression and instrumented fusion were done with substantial bone grafting. Vancomycin powder was placed deep into the posterior wound and a multilayer closure was done. After completing 24 hours of IV cefazolin postoperatively, the patient was then transitioned to empiric oral Augmentin until cultures would result. On postoperative day 3, two of three instrumentation cultures returned positive for CoNS. Infectious disease services were consulted, and he was started on IV vancomycin and oral rifampin for 8 weeks, after which he was transitioned to oral doxycycline for the next 6 months. At the 6-month follow-up, he demonstrated markedly improved lower back and right leg pain, with an increase in his activity and a drastically improved functional capacity, with no signs or symptoms of infection and radiographic evidence of fusion at L5-S1.

## Discussion

Indolent infection is an under-recognized cause of pseudarthrosis of the lumbar spine. Pseudarthrosis can occur because of several reasons, including modifiable and nonmodifiable patient risk factors; however, spine surgeons should always have a suspicion that an infectious cause may be responsible even in the absence of overt signs of infection and normal inflammatory laboratory values. In such cases, low virulent bacteria, such as *C acnes* and CoNS species, should remain high on the differential.

*C acnes* has traditionally been associated with periprosthetic joint infections of the shoulder but can also cause spine infections.^19,20^ The presentation of patients with periprosthetic joint infection secondary to *C acnes* is similar to those who develop spinal pseudarthrosis: often delayed with pain, decreased range of motion, and normal infectious laboratory values.^19^ It has recently gained attention as an increasingly prevalent pathogen in spinal surgical site infections.^7,12,14,21-23^ *C acnes* is likely an under-recognized cause of pseudarthrosis of the lumbar spine due to the need for multiple intraoperative cultures, extended culture time for positive results, and nonstandard hospital methodologies for the aseptic workup.^6^ Steinhaus et al^13^ recently examined presumed aseptic revision spinal surgeries and found both male sex and a diagnosis of pseudarthrosis-predicted positive cultures, which were most commonly positive for *C acnes*. Shifflet et al also examined presumed aseptic revision surgery and found that in cases with positive cultures, *C acnes* grew in 48.9% and CoNS in 11.1%. The average time to positive culture for *C acnes* was 6.1 days, reinforcing the need for extended incubation of cultures even in presumed aseptic cases.^14^ Burkhard et al^6^ found that 10.2% of cases were culture positive in revisions for presumed aseptic pseudarthrosis, with the results of *C acnes* 46.2% and CoNS 38.5% of the time. In patients without clinical signs of infection, Hu and Lieberman^22^ found that *C acnes* was the most frequently identified pathogen of all the implants removed, comprising 46.7% of these infected implants.

When revision is required, surgical approaches include posterior only, combined anterior and posterior, and anterior only. Albert et al^24^ recommended a combined anterior and posterior approach because of the ability to enhance the chance of fusion anteriorly and posteriorly. A recent meta-analysis of all TLIF procedures found a fusion rate of 93.3%.^25^ However, Safaee et al^18^ recently published their results of using an anterior approach to remove TLIF cages for pseudarthrosis, demonstrating that ALIF rescue was 96.6% successful with only a 2.4% chance of additional revision surgery in 84 patients who were able to simultaneously have the TLIF removed from this anterior-only approach. Similarly, Yun et al^17^ described a 9-patient case series with salvage ALIF after TLIF or PLIF, with all patients having successful fusion and improved clinical outcomes.

The anterior approach to the retroperitoneal space is the most powerful revision approach for a number of reasons. First, the epidural scar tissue from the posterior approach used to implant the TLIF during the index surgery can be avoided altogether, decreasing the chance of creating iatrogenic injury to the dura and neural elements during exposure and cage retrieval. The anterior access, if not previously operated on for unrelated abdominal pathologies, provides virgin tissue free of adhesions and obscured by scar, thereby creating a safer environment to operate and achieve the same objectives without the risk of causing neural damage from undue manipulation in a revision setting. A lateral approach can be considered above L5/S1 but may prove to be more challenging in retrieving a TLIF cage that was originally implanted in a perpendicular trajectory. The surgical window is much smaller and may preclude insertion of accessory instruments in helping to remove well-fixed cages. Additional end plate damage may also be incurred. Second, pseudarthrosis from infection still needs to be addressed, and an anterior approach can provide a larger working area to achieve both a more comprehensive débridement of the infected disk space and theoretically provide a larger scaffold for which a fusion can be achieved. In addition, fusion can be augmented through the anterior approach with an adjunctive use of rhBMP-2. The use of rhBMP-2 for lumbar fusion is only FDA approved for ALIF. Several studies have demonstrated equal or superior results with the use of rhBMP-2 over an iliac crest autograft.^26,27^ Therefore, in an attempt to overcome a previously failed fusion, the use of rhBMP-2 is a welcome adjunct to achieve this. However, there are certainly reservations in doing so in the setting of an active infection. Despite this, multiple studies have investigated this surgical dilemma. Allen et al^28^ reported a case series of patients in which 14 patients underwent circumferential fusion with the use of rhBMP-2. They were able to discern that rhBMP-2 use was safe and effective for solid fusion in their series with a two-year follow-up. Recently, Yaw Tee et al^29^ conducted a retrospective study examining reinfection rates and rates of revision surgery in cohorts with and without the use of BMP in the setting of active infection and found that there was no increased rate of either outcome in using BMP. Moreover, in a consensus statement by Walker et al,^30^ rhBMP-2 was deemed safe and efficacious for use in the setting of vertebral osteomyelitis/diskitis based on the current available literature. Given these reported trends in spine surgery, we elected to use BMP as a safe adjunct for fusion through an anterior lumbar approach.

In the illustrative cases in this study, both patients had cultures return positive shortly after surgery and appropriate antibiotics were started. At our institution, if patients are able to be discharged before final cultures have resulted, patients will complete 24 hours of perioperative antibiotics and then will be transitioned to empiric oral antibiotics, typically Augmentin. Once cultures were ensured to be negative, these empiric antibiotics could be safely discontinued. Typically, laboratories were asked to hold the cultures for at least 2 weeks, according to the most recent shoulder surgery literature recommendations, which are notoriously affected by *C acnes* infections.^31,32^

There are several possible limitations to our study: The retrospective nature of the investigation has the potential to generate biases inherent to retrospective studies. The single-center design of the study and small patient sample size limit the ability to generate more generalized conclusions, as does lack of a long-term follow-up in the discussed patients. Regardless, this case series examines an under-investigated cause of pseudarthrosis and outlines an effective method of management for failed lumbar fusion due to indolent infection.

Pseudarthrosis after TLIF or PLIF in an otherwise asymptomatic patient should raise suspicion for indolent infection of the implant. Management should consist of revision surgery, optimally through an anterior lumbar approach to promote ease of cage removal, wide débridement, and to optimize alignment and the surface area available for revision fusion, all while circumventing a scarred posterior surgical field.
